# *Tetraselmis chuii* as a Sustainable and Healthy Ingredient to Produce Gluten-Free Bread: Impact on Structure, Colour and Bioactivity

**DOI:** 10.3390/foods9050579

**Published:** 2020-05-04

**Authors:** Maria Cristiana Nunes, Isabel Fernandes, Inês Vasco, Isabel Sousa, Anabela Raymundo

**Affiliations:** LEAF—Linking Landscape, Environment, Agriculture and Food, Instituto Superior de Agronomia, Universidade de Lisboa; Tapada da Ajuda, 1349-017 Lisbon, Portugal; icxfernandes@gmail.com (I.F.); inesfsvasco@gmail.com (I.V.); isabelsousa@isa.ulisboa.pt (I.S.); anabraymundo@isa.ulisboa.pt (A.R.)

**Keywords:** gluten-free bread, microalga *Tetraselmis chuii*, rheology, texture, colour, antioxidants, phenolics

## Abstract

The objective of this work is to increase the nutritional quality of gluten-free (GF) bread by addition of *Tetraselmis chuii* microalgal biomass, a sustainable source of protein and bioactive compounds. The impact of different levels of *T. chuii* (0%—Control, 1%, 2% and 4% *w*/*w*) on the GF doughs and breads’ structure was studied. Microdough-Lab mixing tests and oscillatory rheology were conducted to evaluate the dough´s structure. Physical properties of the loaves, total phenolic content (*Folin-Ciocalteu*) and antioxidant capacity (DPPH and FRAP) of the bread extracts were assessed. For the low additions of *T. chuii* (1% and 2%), a destabilising effect is noticed, expressed by lower dough viscoelastic functions (G’ and G’’) and poor baking results. At the higher level (4%) of microalgal addition, there was a structure recovery with bread volume increase and a decrease in crumb firmness. Moreover, 4% *T. chuii* bread presented higher total phenolic content and antioxidant capacity when compared to control. Bread with 4% *T. chuii* seems particularly interesting since a significant increase in the bioactivity and an innovative green appearance was achieved, with a low impact on technological performance, but with lower sensory scores.

## 1. Introduction

This study is part of Algae2Future project, that intends to explore the microalgae potential to be a low-carbon/nitrogen-footprints healthy food ingredient. These photosynthetic unicellular organisms have a huge importance in terms of the carbon dioxide mitigation [1] and nitrogen balance [2]. Moreover, microalgae are considered to be one of the most promising sources of functional food ingredients since their natural encapsulated bioactive compounds to promote important health benefits [3,4]. In the near-future context of food shortage and urge for sustainability, when the rate of population increase will be higher than increase of food production, caused by several environmental, social and economic factors, the use of alternative or under-exploited sources of protein is a very important issue. Microalgae are exceptional protein resources with potential to become a staple food for consumers all over the planet [1,5,6].

When considering microalgae as food for the future, it is also important to highlight that its incorporation into food is a challenge. There is a technological limit of microalgae incorporation, resulting from its impact on the food structure, that can be followed by a change on the rheology behaviour [7,8,9]. The introduction of microalgae biomass imparts changes in foods structure, but also in colour and flavour. Consumers are very sensitive to the changes in sensory characteristics, which induces limitations on the level of microalgae incorporation. *Tetraselmis chuii* is a green microalga approved by EFSA (Regulation UE 2017/2470) which has a high protein content, that is an important requirement to be used in bread with a specific nutritional profile. In the last few years, several works have been developed about the incorporation of microalgae into food. Many innovative food systems enriched with algae have been proposed, namely pasta [10,11,12,13], cookies [14,15,16] and wheat bread [17,18].

Bread is a staple food with specific characteristics in terms of the development of a cohesive and elastic dough structure. Since gluten confers unique rheological properties to yeast-leavened baked products, the absence of gluten is a major technological drawback. The combination of structural ingredients, including starches, gluten-free (GF) flours, hydrocolloids, proteins and additives, to develop an alternative to gluten dough´s structure has been widely tested [19,20,21]. Therefore, the addition of microalgae with high protein content can be important for the development of GF bread. In addition, nutritional benefits can be achieved by the addition of microalgae, and this is particularly important in GF bread, since celiac patients have nutritional deficiencies due to their absorption limitations.

Gluten-free is a current hot topic in the food industry. Consumption of GF products, and particularly bread, has increased considerably in recent times. This is due not only to the increase of celiac disease, but also to an increase in other gluten-related disorders [21]. Now, an increasing number of consumers, who have not been diagnosed with celiac disease, are cutting gluten from the diet and GF products are aligned with the top ten food trends.

Research on GF products with algae is scarce. From our knowledge, the only study focused on the technology and nutritional properties of GF bread supplemented with a cyanobacteria (*Arthrospira platensis*, known as Spirulina) was published by Figueira et al. [22], and more recently Rózylo and co-workers [23] determined the effect of brown macroalgae (*Ascophyllum nodosum*) on GF breads and observed increased antioxidant activity. In GF fresh pasta, the seaweed *Laminaria ochroleuca* showed promising potential to be used and similar mechanical and texture characteristics to the reference sample were achieved [12,13].

In the present study, a formulation based on buckwheat, rice flours and potato starch was enriched with *Tetraselmis chuii* biomass. Doughs prepared with the addition of microalgal biomass (1%, 2% and 4% *w*/*w*) were characterized using mixing tests in a MicrodoughLab and by oscillatory tests in a controlled-stress rheometer. The amount of water required to yield the same dough consistency of the previously optimized control-formulation was determined by the mixing test for the formulations enriched with microalgal biomass. Texture, volume and colour properties were used to evaluate the impact of different levels of *T. chuii* on the bread quality. Total phenolic content and reduction power of the bread extracts were assessed.

## 2. Materials and Methods

### 2.1. Raw Materials

Tetraselmis chuii was produced and collected by A2F partners in NORCE/UiB (Bergen, Norway). Cell wall disruption was applied to promote a controlled release of the active biocompounds, as it was recently proven in a previous study [18]. Fresh biomass was pre-processed by bead milling mechanical treatment in the pilot unit of NOFIMA and freeze-dried. It was determined to have high content of protein and also an important content of bioactive compounds: 47.7% protein, 11.4% lipids with 3.6% EPA, 2.4% starch and 2.3% salt [24].

Samples were prepared with the following flours: buckwheat flour (Próvida, Mem Martins, Portugal), rice flour (Espiga, Alcains, Portugal), potato starch (Globo, Seixal, Portugal), dried yeast (Fermipan®, Setúbal, Portugal), hydroxipropylmethylcellulose (HPMC) as a gelling agent (WellenceTM 321, Dow, Bomlitz, Germany), commercially available sugar, sunflower oil, salt, and water. The flours and HPMC were kindly supplied for free, except yeast, sugar, oil and salt that were purchased from local market. The commercial GF mix (Schär, Burgstall, Italy) has the following ingredients: corn starch, flax flour 12%, buckwheat flour 8%, pea bran, rice bran, apple fibre, sugar, guar seed flour, salt.

### 2.2. Preparation of the Samples

To compare the performance of GF breads with *T. chuii*, 2 controls were set up: a commercial GF mix to bake bread at home and a blank test without microalgae, called Control dough. The commercial GF bread was prepared from the mix, following the recommendations described on the product label.

GF breads with *T. chuii* (1.0 g, 2.0 and 4.0 g of *T. chuii*/100 g of flours + *T. chuii*) were prepared according to a previously optimised method [25]. This formulation, based on buckwheat and rice flours and potato starch, was tested using the specific volume and crumb firmness of the resulting breads as responses. Hydroxypropylmethylcellulose (HPMC), which is commonly used in GF breads, was used as a thickening agent, binding water and increasing doughs viscosity. The formulations studied are summarised in Table 1.

Ingredients were mixed in a thermoprocessor equipment (Bimby—Vorwerk, Carnaxide, Portugal), initially to activate the yeast, by adding water, yeast and sugar for 2 min at 27 °C, at velocity 1. Following, the other ingredients were added and mixed for 10 min in a dough mixing program (wheat ear symbol). The resulted dough was placed in a fermentation chamber Arianna XLT133 (Unox, Cadoneghe, Italy) for 50 min at 37 °C. For breadmaking tests, the dough was baked in an electric oven Johnson A60 (Johnson & Johnson, New Brunswick, NJ, USA) at 180 °C for 50 min. Breads were analysed after cooling for 2 h. Three loaves of each formulation were prepared, and all the analysis were performed minimum in triplicate.

The pH values of the doughs ranged from 5.3 to 5.4, respectively for the commercial mix and control-dough, and increased with *T. chuii* incorporation (5.5 in Tc 1%, 5.7 in Tc 2% and 5.9 in Tc 4%).

### 2.3. Mixing Behaviour of the Dough

The Micro-doughLab 2800 (Perten Instruments, Sidney, Australia) was used to investigate the differences in formulation performances and determine the optimum water absorption capacity for each microalgal content (1% to 4% *w*/*w*). Tests were carried out using 4.00 ± 0.01 g mixed flours, at 14% moisture basis, using full formulation. The moisture of the different materials was measured through an automatic moisture analyser PMB 202 (Adam Equipment, Oxford, NJ, USA). Samples and water weights were corrected from flours and microalga moisture content. Standard manufacturer’s protocol “General Flour Testing Method” was used, mixing the samples at a constant 63 rpm speed and temperature of 30 °C for 20 min. The peak value of torque of the optimised control-formulation was used, as a reference, to assess the optimum water absorption for each GF bread formulation with *T. chuii* addition. As a result, mixing curves and dough’s mixing properties were assessed—peak resistance (mN.m), dough development time (s), stability (s) and softening (mN.m).

The amount of water added to the control dough (without microalgal biomass) was 69% on flours mixture basis. This water content was determined in the preliminary assays to produce breads having the best quality, based on bread volume and crumb firmness.

### 2.4. Viscoelastic Behaviour of the Dough

Small amplitude oscillatory shear (SAOS) measurements were performed in a controlled stress rheometer (Haake MARS III, Thermo Fisher Scientific, Waltham, MA, USA) equipped with a UTC-Peltier and fitted with a serrated parallel plate system with 20 mm diameter (PP20) and 1 mm gap (previously optimized for this type of material). After mixing, the dough was shaped into small balls and fermented in the oven at 37 °C, during 50 min. The fermented samples were placed between the plate sensor and the dough surface exposed was coated with paraffin oil to prevent drying, and allowed to rest at 5 °C ± 1 °C for 10 min before testing at same temperature to avoid fermentation during tests. Stress and frequency sweep tests were performed: stress sweep test at 6.28 rad/s (1 Hz) was always performed prior to the frequency sweep, to ensure testing within the linear viscoelastic zone. The viscoelastic properties of the dough were determined from the frequency sweep tests, applying a sinusoidal shear stress of 10 Pa (previously determined linear viscoelastic limit) over an angular frequency range of 0.0628 to 628 rad/s. For each sample, at least three repetitions were performed.

### 2.5. Evaluation of the Bread Texture

Bread texture was characterised using a Texturometer TA.XTplus (Stable Micro Systems, Surrey, UK) equipped with a 5 kg load cell, in a temperature controlled room at 20 ± 1 °C. Bread crumb was measured 2 h after baking by a puncture test using a cylindrical acrylic probe of 10 mm diameter (p/10) at 1 mm·s^−1^ crosshead speed and 10 mm penetration distance. Loaves were sliced by hand, with 20 mm thick. Measurements were repeated at least six times for each bread. Firmness (N) and cohesiveness were the texture parameters used to discriminate different bread samples [26].

### 2.6. Evaluation of the Bread Volume

Volume of the bread was measured using rapeseed displacement method AACC 10-05.01. In order to compare different breads, the same weight of ingredients, in relation to 300 g of flours in mixture with *T. chuii*, was used to prepare all the breads, using the formulations presented in Table 1. All the samples were evaluated, at least, in triplicate.

### 2.7. Evaluation of the Bread Colour

The bread colour was measured using a Minolta CR-400 (Japan) colorimeter with standard illuminant D65 and a visual angle of 2°. The results use the CIELAB system: L*—lightness (0 to 100), a*—greenness to redness (−60 to 60), and b*—blueness to yellowness (−60 to 60). The total colour difference between breads containing the microalgal biomass and the Control sample was calculated using the equation ΔE* = [(ΔL*)^2^ + (Δa*)^2^ + (Δb*)^2^]^1/2^. The measurements were replicated at least six times under artificial fluorescent light using a white standard (L* = 94.61, a* = −0.53, and b* = 3.62).

### 2.8. Evaluation of the Bread Bioactivity

To evaluate the bioactivity of breads, aqueous extracts were prepared by freeze-drying the bread samples and then milling into homogeneous fine powders using an electric blender. Then, 500 mg of each sample was stirred in 50 mL of distilled water using a magnetic bar for 30 min at room temperature, and then filtered by Whatman n° 4 paper. This procedure was repeated in duplicate for each bread. For all the bioactivity analysis, three replicates were performed for each extract, correspondent to six measurements for each bread sample.

The total phenolic content (TPC) of bread extract was evaluated using the method reported by Oktya et al. [27], with some modifications. The aqueous bread extract (300 μL) was added to 1500 μL of 0.1 mol/L *Folin–Ciocalteu* reagent and mixed with 1200 μL of sodium carbonate (7.5%) after 10 min. The mixtures were incubated in the dark at room temperature for 2 h, and then the absorbance was measured at 760 nm in a UNICAM UV4 UV/Vis Spectrometer. For blank testing, the extract was replaced by the same volume of water. The TPC was reported as milligrams of gallic acid equivalents per gram of extract (db).

The scavenging effect of bread extracts was determined using the DPPH (2,2-diphenyl-1-picryl-hydrazyl-hydrate) methodology [28]. Extraction solutions with a volume of 100 μL each were added to 1000 μL (90 μmol/L) of the DPPH solution in methanol, and the mixture was diluted with 1900 μL of methanol. After 1 h in the dark at room temperature, the absorbance was measured at 515 nm. In blank testing, the extract was replaced by the same volume of methanol. The reducing power of the bread extracts was determined using the ferric ion reducing antioxidant power (FRAP) assay [29]. The bread extract (90 μL) was added to 270 μL of water and 2.7 mL of FRAP reagent (2,4,6-tripyridyl-s-triazine (10 mmol/L) with HCl (40 mmol/L), FeCl3 (0.02 mol/L) and acetate buffer (0.3 mol/L pH 3.6) in a ratio of 1:1:10). After 30 min of incubation period in a 37 °C water bath (Thermo Scientific Precision 2864), the absorbance was measured at 593 nm wavelength. For the blank, the extract was substituted by the same volume of water. The mean values were reported as milligrams of ascorbic acid equivalents per gram of extract (db).

### 2.9. Sensory Evaluation

Hedonic sensory evaluation was performed for breads with 1 and 4% of T. chuii, as well as for control sample, using an untrained panel of 32 consumers, randomly chosen among the staff and students from the Instituto Superior de Agronomia Food Science Department, 14 males and 18 females, with ages between 16 and 65. The three breads were analysed in terms of colour, smell, taste, texture and general acceptance using a 5-point hedonic scale from “very unpleasant” (1) to “very pleasant” (5). The assays were conducted in a standardized sensory analysis room, according to the standard EN ISO 8589: 2007.

### 2.10. Statistical Analysis

The analysis of variance (one-way ANOVA) of the experimental data was performed using Origin Pro 8.0 software (OriginLab Corporation, Northampton, MA, USA), followed by Tukey’s test. Correlation analysis was performed by using STATISTICA (version 10.0, StatSoft, Hamburg, Germany) and the function Bivariate Scatterplot. The significance level was set to 95% (*p* < 0.05).

## 3. Results and Discussion

### 3.1. Impact of Tetraselmis chuii Addition on the Dough Rheology

#### 3.1.1. Empirical Methods—MicrodoughLab

**Figure 1:**
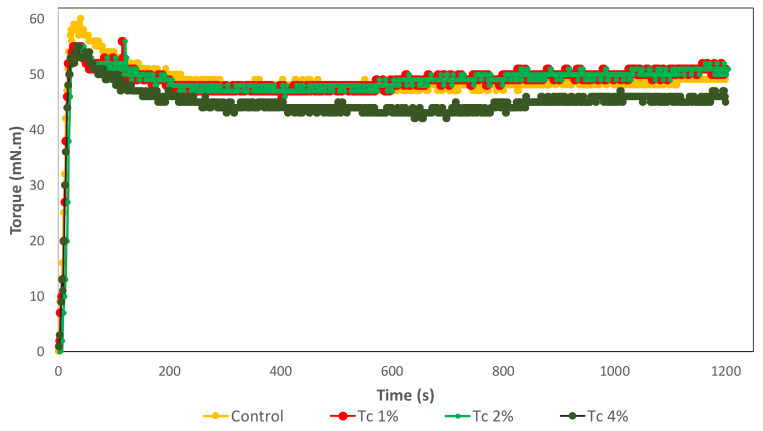
Mixing curves obtained from the Micro-doughLab of Control and GF doughs with *T. chuii* Tc 1%, Tc 2% and Tc 4%.

The mixing curves obtained from the MicrodoughLab are represented in Figure 1, where resistance to mixing is measured as torque. The manufacturer’s protocol “General Flour Testing Method”, at slow speed, was adopted. GF bread doughs have a completely different composition and structure from the wheat dough. These GF systems are mainly composed of starch granules embedded in a matrix composed of proteins and other hydrocolloids, but without a gluten network [21]. GF doughs are adhesive and have poor mixing properties. The mixing curves showed a high initial torque as water was hydrating the flours, followed by torque decrease and unstable mixing curves were obtained, without a peak development as observed in wheat doughs mixing curves. Although this test was developed for wheat dough, and aims a targeted peak of 100 mN.m torque, for an optimum consistency, it is not possible to use this value in GF doughs. Therefore, different approaches have been used to determine the water absorption level of GF formulations. Some researchers carried out preliminary assays to optimise the amount of water necessary for bread making process based on the specific volume of the produced bread [30,31] and crumb hardness of the resulting breads [30]. Others, used the farinograph mixer in order to achieve an optimal dough consistency of 200 BU [32] or 500 BU—Brabender units [33], dough consistency checked by the texturometer equipped with back extrusion rig, using a reference value of firmness [34], comparison of complex viscosity (η*) values vs. frequency, taking a soft wheat flour dough which gave a peak at 500 BU as a reference [35], etc.

Our proposed methodology is to assess optimum water absorption values (amount of water needed to achieve target peak torque, corrected to 14% moisture basis) for the formulations containing microalgal biomass in order to achieve a peak of 56 nN.m ± 5 mN.m. This peak value corresponds to a control-dough (same GF formulation without microalgae) with good bread baking characteristics, previously optimised by testing different water hydrations (data not presented). The technological parameters obtained from the mixing curves are presented in Table 2. For the microalgae contents considered in the present study (1 to 4% *w*/*w*) and 69% of water absorption, similar peak values were obtained, without being necessary to adjust the water content of microalgal-containing formulations. From this empirical rheology test, it was concluded that *T. chuii* addition had no significant (*p* < 0.05) impact on the rheology parameters extracted from de mixing curves: optimum water absorption, peak development time, stability and softening.

The GF mix was not evaluated using Micro-doughLab since water hydration followed the recipe indicated on the label of this commercial product.

#### 3.1.2. Fundamental Methods—Small Amplitude Oscillatory Shear Measurements (SAOS)

**Figure 2:**
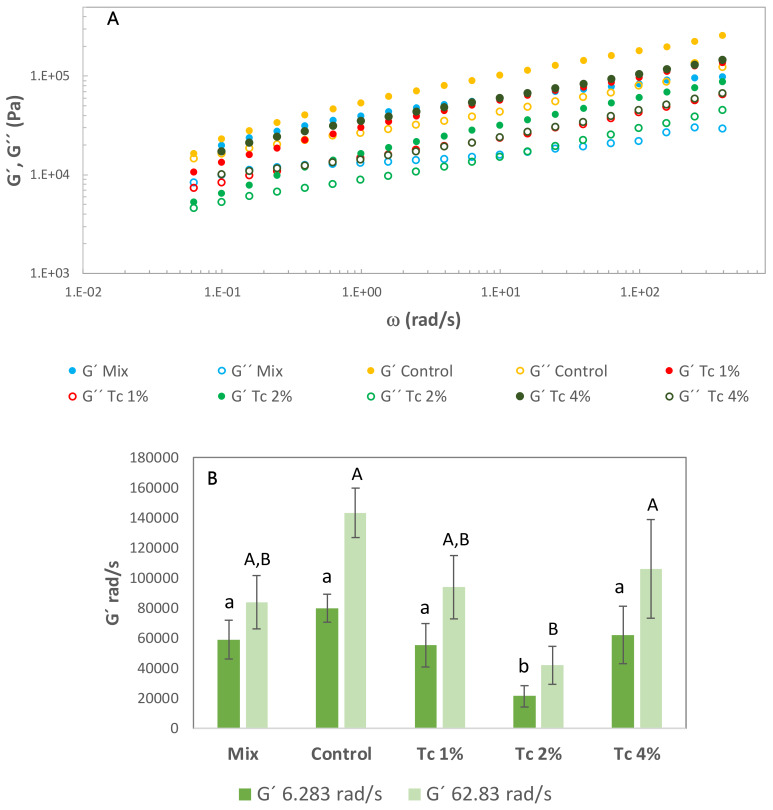
(**A**) Mechanical spectra and (**B**) values of G’ at 6.283 rad/s (1 Hz) and 62.83 rad/s (10 Hz) obtained after GF dough fermentation. G’ (storage modulus—filled symbol), G’’ (loss modulus – open symbol). Mix, Control and GF dough with *T. chuii* Tc 1%, Tc 2% and Tc 4%. Error bars indicate the standard deviations from the repetitions. Different letters correspond to significant differences (*p* < 0.05).

Frequency sweeps were performed to evaluate the impact of *T. chuii* biomass addition on dough structure, after fermentation (Figure 2). For each sample, the test was performed in triplicate, and the most representative curve for each sample is presented. Doughs have a viscoelastic behaviour with G’ > G’’, for the whole range of frequencies studied, showing a destructuring effect, resulting from the microalgae addition, that was observed at 1% and 2% levels. However, for higher Tc incorporation, 4%, an increase of G’ values was observed. For the 4% *T. chuii* dough, the values of the elastic modulus (G´ at 6.283 rad/s and 62.83 rad/s) are significantly (*p* < 0.05) higher than for 2% *T. chuii*, and are similar to the commercial mix, control-sample and 1% *T. chuii*. These values correspond to a structure recovery at 4% level of microalgae addition.

The frequency dependence of G’ (elastic modulus) and G’’ (viscous modulus) could be described by the power law Equations (1) and (2):

G’ = a’ 𝛚^b’^ (1)

G’’ = a’’ 𝛚 ^b’’^ (2)

Values of a and b are determined by performing a linear regression on log G’ and G’’ versus log frequency, where a’ and a’’ are the y-intercepts and b’ and b’’ are the slopes of the resulting line [7]. According to a’ and a’’ values, Tc 2% dough presented a lower level of structure, showing the lower a’ value and the highest value of b’ (Table 3).

Flours from grains without gluten, such as rice, and from pseudocereals, such as buckwheat, have been used as ingredients to produce GF breads. Addition of pseudocereals considerably improves dough viscosity and the texture, volume and nutritional quality of GF products [20,36] and could lead to improved anti-staling properties [37]. To our knowledge, studies relating the impact of microalgae in GF dough rheology have not been published so far. For wheat bread, Nunes et al. [18] have studied the impact of microalgae cell disruption pretreatment on the dough rheology and bread bioactivity and found that 1% *Chlorella vulgaris* has a negative impact on the dynamic viscoelastic properties of the wheat dough.

The structure of complex GF dough systems is mainly accounted by starch and HPMC which could entrap the air. When small amounts of protein are added, coming from *T. chuii*, a destabilisation of this structure can be noticed (Figure 2), mainly for the sample with 2% of microalgal incorporation, however there is a structure recovery for 4% addition. It looks like microalgae elements at first disrupt the structure formed by starch granules embedded in amylose matrix reinforced by HPMC long rods, this can probably happen by depletion flocculation by antagonism with protein macromolecules from *T. chuii*, up to a certain concentration. For higher levels (4%), the proteins should take the lead in the structure network, playing an important role by replacing the former structure, contributing to a new matrix, dominated by protein interactions, where the starch granules and HPMC will be embedded with some compatible reinforcement of the amylose released from starch granules. This structure is different from the previous one, more compact, as it can be seen by the smaller size of the crumb alveoli.

### 3.2. Impact of Tetraselmis chuii Addition on the Breadmaking Properties

**Figure 3:**
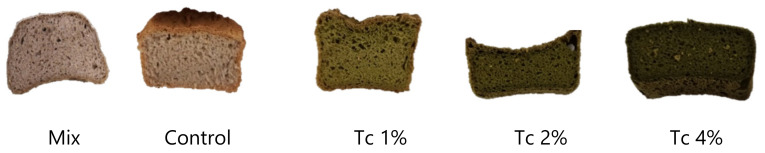
General aspect of the GF breads prepared with commercial mix, control and 1, 2 and 4% (*w*/*w*) *T. chuii* gluten free breads.

The impact of *T. chuii* incorporation in the GF bread shape and colour can be accessed through Figure 3.

**Figure 4:**
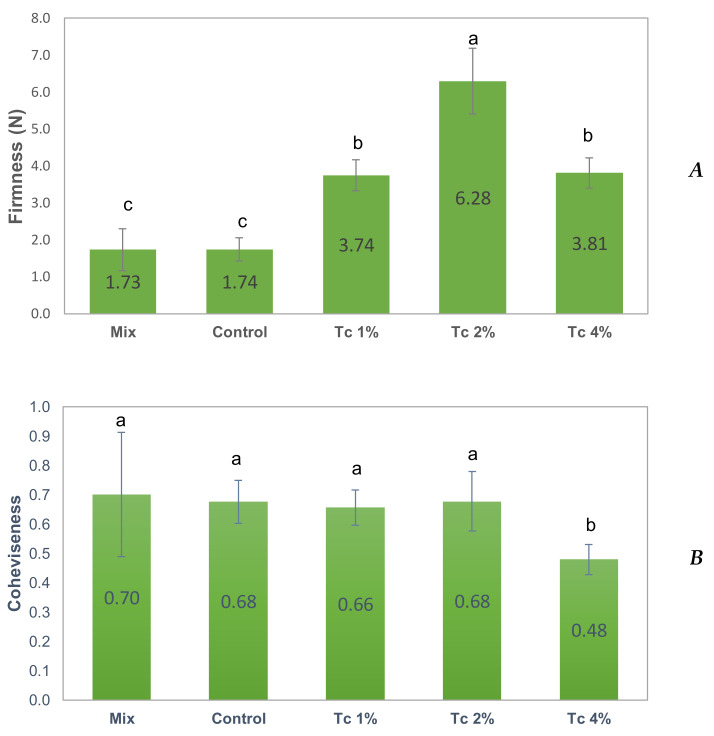
Firmness (**A**) and cohesiveness (**B**) values of GF doughs with *T. chuii* obtained by the texturometer. Mix, Control and GF dough with *T. chuii* Tc 1%, Tc 2% and Tc 4%. Error bars indicate the standard deviations from the repetitions (*n* = 6). Different letters in the same graph correspond to significant differences (*p* < 0.05).

To study the relation between overall bread quality parameters, raw materials and dough properties, the texture and the volume of bread loaves were evaluated. For bread crumb differing in composition, crumb firmness increased with microalgal addition until 2% *T. chuii* (Figure 4) and there was a negative impact on loaf volume (Table 4). However, at 4% level, there was an increase of the bread volume and a significant (*p* < 0.05) reduction of bread crumb firmness comparing to 2%, not differing from 1% *T. chuii* bread. Nevertheless, this formulation with 4% of microalgal biomass presented a significant (*p* < 0.05) lower cohesiveness when compared to all the others, including the control-bread and the bread prepared with a commercial mix.

Starch gelatinization plays an important role in GF formulations, due to the ability of starch to form a matrix in which gas bubbles are entrapped [38]. Incorporation of non-gluten proteins in the formulation, even at lower amounts, may be essential in achieving final product volume [30,39,40]. Considering the present results, it is possible to conclude that at low contents of *T. chuii* incorporation, microalgal protein induces a destabilisation of the network formed by starch and HPMC. This is revelled by a significant reduction of bread volume (the bread becomes more compact), as well as a significant increase of firmness. However, for the highest protein content of 4%, microalgal protein plays an important role on the GF dough structure, increasing the capacity to retain the gas bubbles.

In what crumb colour is concerned (Table 4), it is possible to conclude that there was a reduction in lightness (L*) and an increase in greeness (a*, in modulus), comparing to commercial mix and control breads. In respect to yellowness (b*), there was a significant (*p* < 0.05) decrease from control (19.2) to 1% *T. chuii* bread (16.2), increasing with 2% of algae (27.1) and decreasing again for 4% *T. chuii* (22.2). These results are related to the pigments of this *Chlorophyceae* green algae, namely chlorophylls, carotenoids (such as fucoxanthin and β-carotene) and α-tocopherol (Vitamin E) [41]. The total colour difference ΔE* between control and *T. chuii* crumbs was higher than 64, despite the small differences between 2% and 4% *T. chuii* bread colours. Several authors consider that the human eye can differentiate colours when the total colour difference ΔE* > 5 [42], therefore big colour changes were obtained when breads were enriched with microalgal biomass. From these results, it seems that *T. chuii* is contributing to a darkening effect, as it is evident by visual observation (Figure 3) and through the instrumental colour parameters. As will be described in Section 3.5., these colour characteristics are not well appreciated by the sensory panel.

### 3.3. Correlations between Breadmaking Properties and Dough Rheology

**Figure 5:**
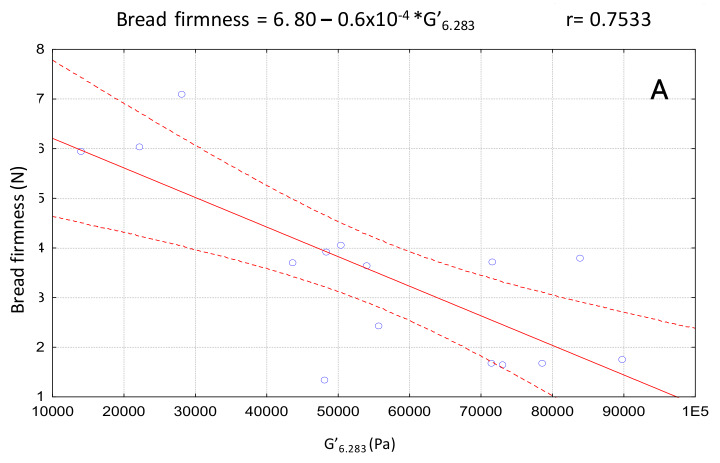

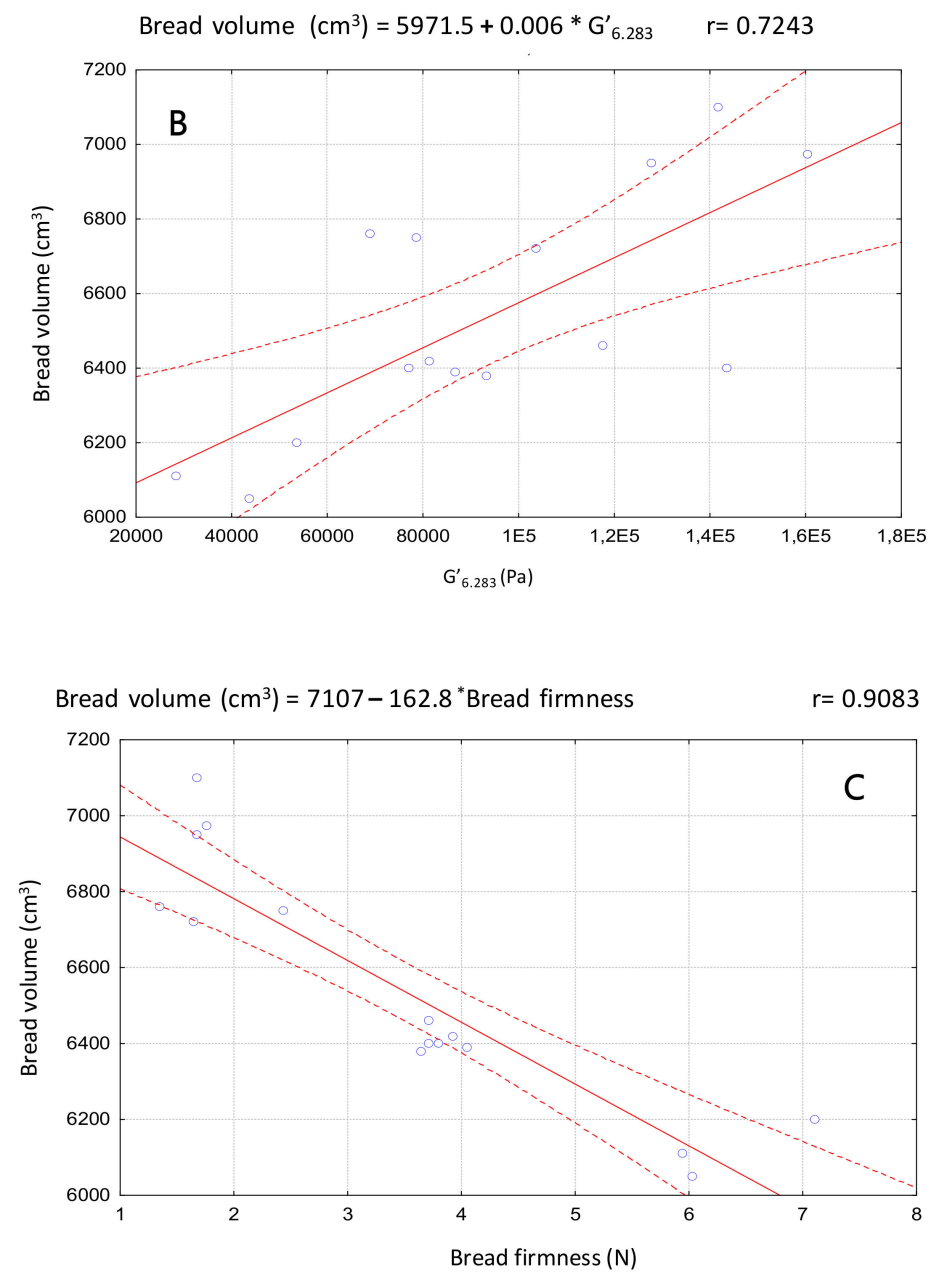
Mathematical correlations between GF breadmaking properties and dough rheology (*p* < 0.05). (**A**) Bread firmness and G’_6.283 rad/s_; (**B**) Bread volume and G’_6.263 rad/s_; (**C**) Bread volume and bread firmness.

Using correlation analysis, the relationships between dough rheology and breadmaking performance of breads with different amounts of *T. chuii* incorporation were obtained. In Figure 5, one can see the bivariate scatterplots of significant (r > 0.70) dependences - bread firmness vs elastic modulus (G’_6.283 rad/s_), bread volume vs elastic modulus (G’_6.283 rad/s_), and bread volume vs bread firmness. High G’ values are related with greater elastic contribution and this is negatively correlated with the bread firmness (softening effect), followed by a bread volume increase. As expected, bread volume presented a negative correlation with bread firmness.

Similar results were described by Martínez & Gómez [43], showing that viscoelastic properties of several GF doughs strongly influenced the bread volume and crumb texture. Elgeti et al. [44] referred that the rheology of starch-based dough systems influences the level of aeration during mixing and baking.

### 3.4. Impact of Tetraselmis chuii Addition on the Bread Bioactivity

Total phenolic content and antioxidant capacity (DPPH and FRAP) were evaluated only for the control and 4% *T. chuii* breads, considering the previous results and the objective of achieving high levels of microalgae incorporation.

**Figure 6:**
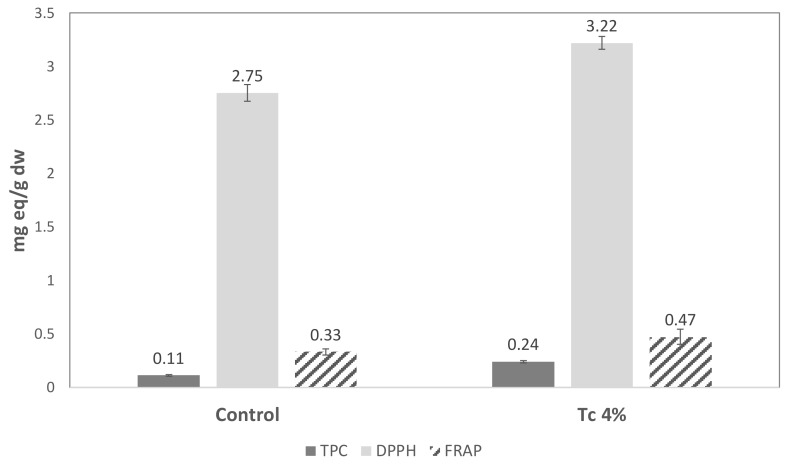
Total phenolic content (mg·g^−1^ gallic acid equivalents), antioxidant capacity measured using the DPPH assay (2,2-diphenyl-1-picryl-hydrazyl-hydrate; mg.g^−1^ ascorbic acid equivalents) and FRAP assay (ferric ion reducing antioxidant power; mg.g^−1^ ascorbic acid equivalents) of GF breads enriched with 4% (*w*/*w*) of *T. chuii* biomass in comparison with Control bread. Error bars indicate the standard deviations of the repetitions (n = 3).

The addition of the microalgal biomass at 4% (*w*/*w*) resulted in a significant (*p* < 0.05) increase in the total phenolic content (TPC) (Figure 6), which was 0.11 mg·g^−1^ gallic acid equivalents in the Control bread and 0.24 mg·g^−1^ in the bread containing *T. chuii*. TPC values were lower than the values reported for GF breads with brown microalgae [23] and may be a result of the formation of protein-phenolic complexes [23,45].

The antioxidant capacity was tested using the DPPH and FRAP methods. Compared with the control-bread (2.75 mg·g^−1^ and 0.33 mg·g^−1^ ascorbic acid equivalents obtained by DPPH and FRAP, respectively), the incorporation of microalgal biomass led to a significant (*p* < 0.05) increase in the antioxidant capacity of the breads (3.22 mg·g^−1^ and 0.47 mg·g^−1^ ascorbic acid equivalents). Even upon baking, the antioxidant activity of these breads is interesting. Some other authors reported similar results, Rózylo et al. [23] determined the effect of brown macroalgae on GF bread and observed increased FRAP activity, and higher FRAP antioxidant capacity was found by Nunes et al. [18] in wheat breads enriched with *Chlorella vulgaris*.

### 3.5. Sensory Evaluation

**Figure 7:**
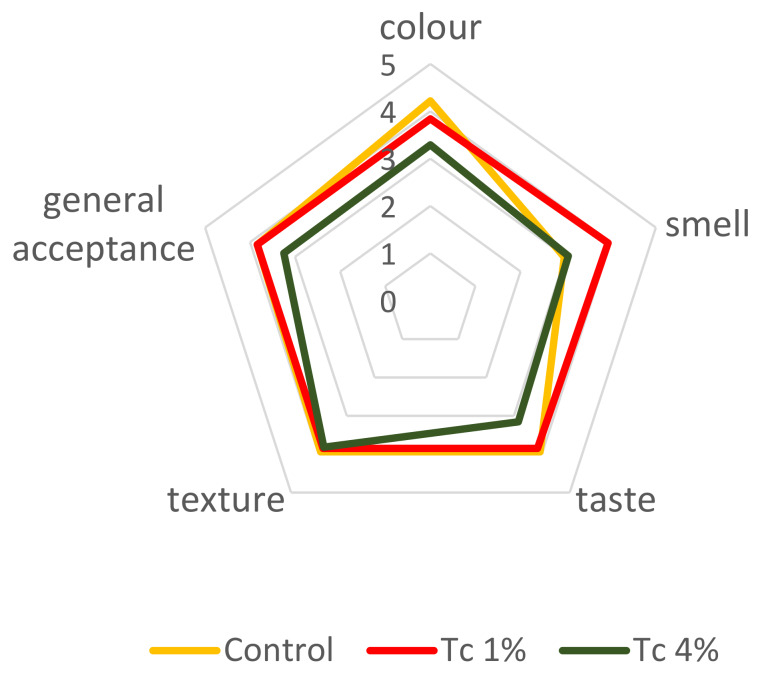
Responses of the sensory analysis panel tasters (*n* = 32) regarding breads enriched with 1% and 4% *T. chuii*, as well as the control sample. 1—“very unpleasant”, 2—“unpleasant”, 3—“indifferent”, 4—“pleasant”, 5—“very pleasant”.

Sensory analysis assays were carried out with *T. chuii* microalgal GF breads, at 1% and 4% incorporation level, in comparison to control. Figure 7 represents the average scores of the sensory parameters as evaluated by the non-celiac panel.

Panellists preferred control bread and bread with 1% *T. chuii*, both with a general acceptance of 3.84, compared to 3.25 of 4% *T. chuii* bread. Texture, colour and taste had similar score for control and 1% *T. chuii* bread. Concerning smell, the tasters preferred the bread with 1% *T. chuii* in relation to control, by a difference of 1.0 point. This can be related to the buckwheat flour, despite the characteristic fishy flavour of *T. chuii* microalgae. Eventhough, for higher microalgal levels, 4% *T. chuii* and control bread had similar scores. As expected for this type of product, for taste, colour and general acceptance, bread with 4% *T. chuii* had lower scores, but not lower than 3, corresponding to “indifferent”. In the comments field of the sensory analysis sheet, some tastes referred the strong fishy flavour of the 4% *T. chuii* bread, but that it could be pleasant to eat with fish meals. Furthermore, this bread could be an interesting alternative for consumers interested in healthy products with an innovative taste and colour. To improve the acceptance by the conventional consumers, educational marketing strategies and formulation enhancements are programmed in the Algae2Future project.

## 4. Conclusions

The mixing and viscoelastic behaviour of GF doughs enriched with *Tetraselmis chuii* was compared with the control formulation. Bread baking performance was also evaluated since GF doughs are complex systems and final bread quality is affected by processing conditions.

*T. chuii* can be used as a natural novel and sustainable ingredient to increase the bioactivity of GF bread based on buckwheat flour, rice flour and potato starch, obtaining an innovative green appearance. Different behaviour was found according with the level of *T. chuii* incorporation. Below 2%, *T. chuii* proteins destabilize the structure developed by starch and HPMC, smaller bread volume was obtained, associated with a more compact crumb and harder properties. However, for higher levels of incorporation (4%), the microalgal proteins with starch and HPMC build up another type of structure, characterised by higher values of the viscoelastic functions (G‘and G’’) producing higher bread volume and a softening effect. This study shows that the structure of 4% *T. chuii* bread is competitive with the control-bread with the advantage of having an improved bioactivity (phenolics and antioxidants) with possible positive impacts on health. The use of *T. chuii* at 4% level is interesting, but low sensory scores postpone its utilization.

## Figures and Tables

**Table 1:** Formulation of gluten-free (GF) samples and respective codes. Control: dough without microalgal biomass; Tc 1%, Tc 2%, Tc 4%: dough with 1%, 2% and 4% (*w*/*w*) *T. chuii*, respectively.

Ingredients (g/100g)	Control	Tc 1%	Tc 2%	Tc 4%
Buckwheat flour	46.0	45.5	45.1	44.2
Rice flour	31.0	30.7	30.4	29.8
Potato starch	23.0	22.8	22.5	22.1
*Tetraselmis chuii*	0.0	1.0	2.0	4.0
Sunflower oil (in relation to flours)	5.5	5.5	5.5	5.5
HPMC (in relation to flours)	4.6	4.6	4.6	4.6
Dried yeast (in relation to flours)	2.8	2.8	2.8	2.8
Sugar (in relation to flours)	2.8	2.8	2.8	2.8
Salt (in relation to flours)	1.8	1.8	1.8	1.8
Water Absorption (14% moisture basis)	69.0	69.0	69.0	69.0

**Table 2:** Parameters obtained from Micro-doughLab mixture curves of Control and GF dough with *T. chuii* Tc 1%, Tc 2% and Tc 4%.

GF Dough	WA (%)	P (mN.m)	DDT (s)	DS (s)	DSO (mN.m)
Control	69.0	56 ± 1.2	48 a	30 a	9 a
Tc 1%	69.0	51 ± 1.7	48 a	44 a	7 a
Tc 2%	69.0	56 ± 3.8	50 a	44 a	7 a
Tc 4%	69.0	51 ± 1.5	48 a	50 a	9 a

Note: WA—water absorption; P—peak resistance; DDT—dough development time; DS—dough stability; DSO—dough softening; PE—peak energy. For each sample, the test was performed in triplicate. Different letters in the same column correspond to significant differences (*p* < 0.05).

**Table 3:** Frequency dependence of G’ and G’’ described by the power-law parameters. Mix, Control and GF dough with T. chuii Tc 1%, Tc 2% and Tc 4%. Different letters in the same column correspond to significant differences (*p* < 0.05). *R*^2^ > 0.91.

GF Dough	a’	b’	a’’	b’’
Mix	38397 ^a^	0.182 ^d^	13942 ^a,b^	0.130 ^a^
Control	43446 ^a^	0.289 ^a,b^	23896 ^a^	0.239 ^a^
Tc 1%	31724 ^a,b^	0.267 ^b,c^	15642 ^a,b^	1.497 ^a^
Tc 2%	11639 ^b^	0.318 ^a^	6875 ^b^	0.268 ^a^
Tc 4%	37213 ^a^	0.249 ^c^	17555 ^a^	0.223 ^a^

**Table 4:** Bread volume and colour parameters obtained for bread crumb. Mix, Control and GF dough with *T. chuii* Tc 1%, Tc 2% and Tc 4%. Different letters in the same column correspond to significant differences (*p* < 0.05).

GF Bread	Volume (cm^3^)	L*	a*	b*	ΔE*
Mix	674 ^b^	89.3 ^a^	8.5 ^a^	24.0 ^a,b^	_
Control	701 ^a^	94.6 ^a^	1.4 ^b^	19.2 ^c^	_
Tc 1%	642 ^c^	38.9 ^b^	−1.1 ^c^	16.2 ^d^	64
Tc 2%	612 ^d^	29.8 ^b,c^	−1.3 ^c^	27.1 ^a^	73
Tc 4%	640 ^c^	25.7 ^c^	−0.3 ^c^	22.2 ^b,c^	77
